# Spanish version of the Oral Health Impact Profile (OHIP-Sp)

**DOI:** 10.1186/1472-6831-6-11

**Published:** 2006-07-07

**Authors:** Rodrigo Lopez, Vibeke Baelum

**Affiliations:** 1 Department of Community Oral Health and Pediatric Dentistry, Royal Dental College, Faculty of Health Sciences, University of Aarhus, Aarhus, Denmark

## Abstract

**Background:**

The need for appraisal of oral health-related quality of life has been increasingly recognized over the last decades. The aims of this study were to develop a Spanish version (OHIP-Sp) of the Oral Health Impact Profile and to evaluate its convergent and discriminative validity, and its internal consistency.

**Methods:**

The original 49-items OHIP was translated to Spanish, revised for understanding and semantics by two independent dentists, and then translated back to English by an independent bilingual dentist. The data originated in a cross sectional study conducted among high school students from the Province of Santiago, Chile. The study group was sampled using a multistage random cluster procedure yielding 9,203 students aged 12–21 years. All selected students were invited to participate and all filled a questionnaire with information on socio-demographic factors; oral health related behaviors; and self-reported oral health status (good, fair or poor). From this group, 9,163 students also accepted to fill a detailed questionnaire on socio-economic indicators and to receive a clinical examination comprising direct recordings of clinical attachment levels (CAL) in molars and incisors, tooth loss, and the presence of necrotizing ulcerative gingival lesions.

**Results:**

The participation rate and the questionnaire completeness were high with OHIP-Sp total scores being computed for 9,133 subjects. Self-perceived oral health status was associated with the total OHIP-Sp score and all its domains (Spearman rank correlation). The OHIP-Sp total score was also directly associated with the 4 dental outcomes investigated (Mann-Whitney test) and the largest impact was found for the outcomes, 'tooth loss' with a mean OHIP-Sp score = 13.5 and 'CAL >= 3 mm' with a mean OHIP-Sp score = 13.0.

**Conclusion:**

The OHIP-Sp revealed suitable convergent and discriminative validity and appropriate internal consistency (Cronbach's α). Further studies on OHIP-Sp warrant the inclusion of populations with a higher disease burden; and the use of test-retest reliability exercises to evaluate the stability of the test.

## Background

Oral health-related quality of life (OHRQoL) is an important patient-centered endpoint to consider when assessing the impact of oral diseases in populations and evaluating the professional interventions used in attempt to improve oral health [1-19]. The Oral Health Impact Profile (OHIP) is a questionnaire designed to measure self-reported dysfunction, discomfort and disability attributed to oral conditions [20], and is based on a conceptual oral health model outlined by Locker [21]. The original instrument has 49 items representing 7 domains (functional limitation, physical pain, psychological discomfort, physical disability, psychological disability, social disability, and handicap) and has been shown to be reliable [22-24]; sensitive to changes [5,11,24,25]; and to exhibit suitable cross-cultural consistency [26]. Although the OHIP is available in several languages (Chinese, Finish, French, German, Japanese, Malaysian, Portuguese, Sinhalese, Somalian, Swedish, and Tagalog), a Spanish translation is not available and there are no suitable alternative OHRQoL tools available in Spanish. The aims of this study were to develop a Spanish version of the Oral Health Impact Profile and to evaluate its convergent and discriminative validity, and its internal consistency for use among Chilean adolescents.

## Methods

### Development of a Spanish version of the Oral Health Impact Profile

One of the authors (RL), a Chilean dentist proficient in Spanish and English, translated the 49 items of the original version of OHIP [20] into Spanish. Special attention was given to develop a questionnaire conceptually equivalent to the original version in order to maintain cross-cultural equivalence. The translation was then revised independently by two bilingual dentists, fluent in both Spanish and English, who gave feedback regarding the understanding and semantics of the translation. Following revision, the Spanish version was back-translated to English by an independent bilingual dentist (PS) who had never seen the original version of the OHIP. The back translation (OHIP-Sp) and the original version of OHIP were then compared in order to identify conceptual differences.

### Study group

The data used to validate the OHIP-Sp [see Additional file 1] originated in a cross-sectional study conducted among high school students from the Province of Santiago, Chile. The study group was obtained using a multistage random cluster procedure to select school classes within schools. The sample consisted of 9,203 students aged 12–21 years, distributed in 310 classes from 98 schools. Details about the sampling strategy have been provided elsewhere [27-29]. The study protocol was reviewed and approved by the local ethical committee of the University of Chile and subjects participated on the basis of informed consent. All students were invited to participate in the study and all accepted to fill a brief questionnaire containing information on socio-demographic factors; oral health related behaviors; and self-reported oral health status (rated as good, fair or poor) [27,29]. From the whole study group, 9,163 students accepted to answer a written questionnaire asking detailed information on socio-economic indicators [30] and to participate in a clinical oral examination involving the recording of tooth loss [31], the presence of necrotizing ulcerative gingival lesions (NUG) [28] and clinical attachment level (CAL) in 6 sites per tooth in molars and incisors [27]. A total of 9,155 students also accepted to fill the OHIP-Sp questionnaire. Owing to the young age of the study population, the recall period considered was 'lifetime', just as the response options for each question were dichotomized as 'Yes' or 'No'.

### Missing values and completeness of the OHIP-Sp version

Cognitive disparity and communication problems among the participants may hamper the use of an instrument and seriously affect the results of scoring systems [32]. To circumvent this problem, subjects with more than 5 missing answers in the OHIP-Sp (n = 22) were excluded from further analysis. The burden of OHIP-Sp and the potential difficulties in answering it were evaluated by counting the number of missing answers. In addition, we calculated the % of subjects responding 'No' for each of the 49 items of OHIP-Sp in order to identify items that could be irrelevant for the young study population included in this study.

### Evaluation of the construct validity of the OHIP-Sp

#### Convergent validity

To assess the convergent validity of the OHIP-Sp, we investigated the association between self reported oral health status (good; fair; poor) and the total unweighted OHIP-Sp score, computed by adding the number of items experienced (0–49), as well as each domain score, using Spearman rank correlation. We hypothesized that students who reported good oral health would have lower scores than subjects who reported fair or poor oral health.

#### Discriminative validity

Four dichotomous dental health outcomes were used: A) 'tooth loss', which was considered present if at least one molar or incisor was absent, B) 'CAL ≥ 1 mm', which was present if at least one of the sites recorded had clinical attachment level measurements ≥ 1 mm; C) 'CAL ≥ 3 mm'; and D) 'NUG', which was considered present if at least one interproximal papilla presented with necrotizing ulcerative lesions'. Details on the clinical examinations and the reliability of the recordings have been previously published [27,28,30,33].

To compare the validity of OHIP-Sp in discriminating between groups with and without oral conditions, the mean OHIP-Sp scores were compared between subjects with and without the four oral health outcomes investigated using the Mann-Whitney test. We hypothesized that subjects with poor oral health outcomes would have higher OHIP-Sp scores. Although this is a rather standard procedure in OHIP validation studies [23,34-38], a potential problem may arise when the assessment of discriminative validity of OHIP relies on statistical significance. The situation may be especially critical if the study group is large, because statistical significance may be obtained without the instrument being able to distinguish between groups in a real scenario. In order to explore this possibility, the 'roctab' command of the software Stata [39] was used to obtain receiver operating characteristic curves (ROC) and to calculate the values for the area under the ROC curves [40] for the ability of the total OHIP-Sp score to predict each of the four outcomes studied. The area under the curve is a proportion which can be interpreted as the probability that a randomly selected person with a positive oral health outcome has a higher OHIP-Sp value than a randomly selected person without the oral health outcome [41]. In a post-hoc analysis, ROC curves for the total OHIP-Sp score and more severe clinical attachment level outcomes (CAL ≥ 4, and CAL ≥ 5 mm); and more extensive tooth loss outcomes (≥ 2, ≥ 3, and ≥ 4 teeth) were used to assess whether OHIP-Sp shows higher discriminative validity with more severe and extensive dental outcomes.

### Internal consistency

'*When items are used to form a scale they need to have internal consistency. The items should all measure the same thing, so they should be correlated with one another. A useful coefficient for assessing internal consistency is Cronbach's alpha*' [42].

Internal consistency was assessed for the total OHIP-Sp score and for each of the seven domains, using the Cronbach's reliability coefficient α [43], which is a measure of intercorrelation between possible subsets of items in the instrument. Average inter-item correlation coefficients were obtained for each of the domains of OHIP-Sp, as well as for the total OHIP-Sp score. *'Cronbach's alpha has a direct interpretation. The items in our test are only some of the many possible items which could be used to make the total score. If we were to choose two random samples of k... *(where k is the number of items)*... of these possible items, we would have two different scores each of them made up of k items. The expected correlation between the scores i*s α' [42].

## Results

The comparison between the original OHIP questionnaire and the back translated English version did not reveal conceptual content differences. The participation rate was high (99.9%) and the completeness of the self-answered OHIP-Sp questionnaire was high with about 99% of the students answering at least 44 items and 87.2% of the subjects answering all 49 questions.

OHIP-Sp total scores and domain scores were computed for 9,133 subjects, 12 to 21 years, and evenly distributed by gender. The oral health impacts found in this study group were low, with a mean OHIP-Sp score of 9.7 and mean domain scores ranging between 0.3 for 'social disability' and 3.0 for 'physical pain' (Table 1). The highest oral health impact was observed for the domains 'physical pain', 'functional limitation', and 'psychological discomfort' with mean OHIP-Sp scores 3.0, 2.1, and 1.9, respectively (Table 1).

**Table 1:** Convergence validity.

OHIP-Sp domains and OHIP-Sp score		Self perceived oral health status			
		
	All students (9,133)	Good (n = 2,217)	Fair (n = 5,964)	Poor (n = 950)	r_s_
	mean [95% CI]	Mean [95% CI]	mean [95% CI]	mean [95% CI]	
Functional limitation	2.1 [2.0;2.1]	1.1 [1.1;1.2]	2.1 [2.1;2.2]	3.8 [3.7;3.9]	0.42^#^
Physical pain	3.0 [3.0;3.1]	2.2 [2.1;2.3]	3.1 [3.0;3.1]	4.4 [4.3;4.6]	0.27^#^
Psychological discomfort	1.9 [1.9;1.9]	1.5 [1.4;1.5]	1.9 [1.9;2.0]	2.7 [2.6;2.8]	0.26^#^
Physical disability	0.9 [0.9;0.9]	0.5 [0.5;0.6]	0.9 [0.9;0.9]	1.9 [1.8;2.0]	0.28^#^
Psychological disability	1.1 [1.1;1.1]	0.4 [0.4;0.5]	1.1 [1.1;1.1]	2.6 [2.4;2.7]	0.35^#^
Social disability	0.3 [0.3;0.3]	0.1 [0.1;0.1]	0.3 [0.3;0.3]	0.9 [0.8;0.9]	0.23^#^
Handicap	0.4 [0.4;0.4]	0.2 [0.1;0.2]	0.4 [0.4;0.4]	1.0 [1.0;1.1]	0.24^#^
OHIP-Sp (all items)	9.7 [9.5;9.8]	6.1 [5.9;6.3]	9.8 [9.7;10.0]	17.2 [16.7;17.8]	0.41^#^

Mean scores and Spearman's rank correlation coefficients between OHIP-Sp and its domains, and self-perceived oral health status. (r_s _= Spearman's rank correlation coefficients; # = P-value < 0.001; [95% CI] = 95% confidence interval for the mean).

### Evaluation of the construct validity of the OHIP-Sp

#### Convergent validity

Self-perceived oral health status and OHRQoL were statistically significantly associated with the total OHIP-Sp score and all the domains (Table 1). Correlation coefficients (r_Spearman_) for the association between self -reported oral health status and the different domains ranged between 0.23 for 'social disability' and 0.42 for 'functional limitation'. The coefficient for the association between the total OHIP-Sp score and self-reported oral health status was 0.41 (Table 1).

#### Discriminative validity

As hypothesized, higher OHIP-Sp total score were observed among subjects with the four oral health outcomes investigated. All differences were statistically significant (Table 2). The largest impact was found for the outcomes 'tooth loss', with a mean OHIP-Sp score = 13.5, and 'CAL ≥ 3 mm', with a mean OHIP-Sp score = 13.0 (Table 2).

**Table 2:** Discriminative validity.

**Oral outcomes**	**OHIP Sp score mean [95% CI]**	**Dif [95% CI]**	**P-value**^§^	**ROC**^&^	**[95% CI]**
Lost ≥ 1 tooth loss					
Yes (n = 1,065)	13.5 [13.0;14.1]	4.3 [3.9;4.7]	< 0.001	0.66	[0.64;0.68]
No (n = 8,068)	9.2 [9.0;9.3]				
Lost ≥ 2 teeth loss					
	-	-	-	0.69	[0.66;0.71]
Lost ≥ 3 teeth loss					
	-	-	-	0.71	[0.64;0.80]
Lost ≥ 4 teeth loss					
	-	-	-	0.76	[0.59;0.93]

Presence of NUG					
Yes (n = 616)	11.9 [11.3;12.6]	2.4 [1.8;3.0]	< 0.001	0.59	[0.57;0.62]
No (n = 8,517)	9.5 [9.4;9.7]				

Presence of CAL ≥ 1 mm					
Yes (n = 6,321)	10.2 [10.0;10.3]	1.6 [1.3;1.9]	< 0.001	0.57	[0.55;0.58]
No (n = 2,812)	8.6 [8.4;8.9]				

Presence of CAL ≥ 3 mm					
Yes (n = 409)	13.0 [12.3;13.8]	3.5 [2.8;4.2]	< 0.001	0.64	[0.61;0.67]
No (n = 8,724)	9.5 [9.4;9.7]				
Presence of CAL ≥ 4 mm					
	-	-	-	0.70	[0.64;0.77]
Presence of CAL ≥ 5 mm					
	-	-	-	0.78	[0.68;0.87]

([95% CI] = 95% confidence interval; NUG = Necrotizing ulcerative gingival lesions; CAL = Clinical attachment loss; § = Mann Whitney; & = Area Under the Receiver Operating Characteristic curve; Dif = Difference for the means).

The estimates for the area under the ROC curve obtained for each of the dental health outcomes studied and the total OHIP-Sp score ranged between 0.56 for having CAL ≥ 1 mm, and 0.66 for 'tooth loss' (Table 2).

The ROC curves obtained for the total OHIP-Sp score and increasing severity of clinical attachment loss revealed increasing values for the area under the curve ranging from 0.57 for CAL ≥ 1 mm to 0.78 for CAL ≥ 5 mm (Table 2). A similar result was obtained for increasing extent of tooth loss with values ranging between 0.66 for tooth ≥ 1 tooth, and 0.76 for tooth loss ≥ 5 teeth (Table 2).

### Internal consistency

Internal consistency (Cronbach's α) of the OHIP-Sp was 0.90 and α values for the different domains ranged between 0.48 and 0.76. (Table 3).

**Table 3:** Internal consistency for OHIP-Sp and its 7 domains

Dimensions	Cronbach's α	One-sided 95% confidence interval for α	Average inter-item correlation
Functional limitation	0.58	0.57	0.13
Physical pain	0.67	0.66	0.19
Psychological discomfort	0.48	0.47	0.16
Physical disability	0.63	0.62	0.16
Psychological disability	0.76	0.75	0.34
Social disability	0.68	0.67	0.30
Handicap	0.65	0.64	0.24
OHIP-Sp total score	0.90	0.90	0.16

A total of 8 items (8, 9, 18, 26, 29, 30, 39, 44) were found to impact on less than 5% of the participants and were therefore considered of infrequent for this young population. A closer examination of these items showed that they concern severe oral health related impacts such as eating/digestion impairment, and the use of prostheses, which can be expected to be rather infrequent among young people.

## Discussion

Cross-cultural adaptation procedures are a critical component of the validation process of an instrument to assess OHRQoL and several guidelines can be found for this purpose [32,44,45]. In the present study, the translation process from English to Spanish was straightforward and the comparison between the original OHIP questionnaire and the back translated English version did not reveal conceptual content differences. The equivalent words needed for translation of the questions were not difficult to find, and the grammar structure of the sentences was not difficult to build during the translation process, possibly owing to the fact that English and Spanish share a common Latin background.

Previous studies have shown a low frequency of oral health impacts for young populations such as the present [23]. Moreover, there are drawbacks of using ordinal scales for questionnaire responses, which may make the scale not only instrument-specific, but also sample- and item-specific [46]. To best of our knowledge, there are no studies addressing this issue on adolescents, but the results of a study on the assessment of changes in the quality of life using OHIP on adults [5] suggest that the differences found between groups may be consistent, regardless of the use of dichotomous or ordinal scoring systems. We therefore considered it best for the purpose of the present study to dichotomize the response options for each question into 'Yes' or 'No'. We realize that this approach departs from the common use of Likert-like scales ranging from 'never' to 'very often' in many OHIP studies. This, and the fact that the use of the Oral Health Impact Profile among adolescents has consistently considered only the 14-item versions of the OHIP, and rather different recall periods [23,47-49], makes direct comparisons between studies rather difficult. We are not aware of studies of the effect of different types of response scales on estimates of validity and consistency for the same study group, but the estimates will almost certainly differ.

The interpretation of the study results should also consider the different recall periods used in different studies. To the best of our knowledge, this is the first study considering a lifetime recall period for the administration of the questionnaire among adolescents. The impact of the use of different recall periods has not been addressed in young populations. In a recent study, John et al., [50] applied a German version of OHIP on adults using 3 different recall periods (lifetime, 1 year, 1 month) and found better consistency for the shortest recall period, and a lower impact of oral health for the lifetime recall period.

The mean score values in this study suggest a relatively low impact of oral health in the population studied, similar to the impact reported previously by Soe et al. among Myanmar adolescents with low levels of dental disease [23], and considerably lower than the oral health impact reported in studies comprising minority adolescent populations with higher oral disease burden [49] and adult populations [51,52].

Our finding that 8 items related to eating impairment, use of prostheses, general health, and inability to function were rather infrequent in this adolescent population, indicates that a number of items from the original OHIP representing severe impairment may be irrelevant for adolescents who have only experienced minor oral disease. Our observations suggest that the highest impacts concern some items from the domains representing 'physical pain'; 'functional limitation' and 'psychological discomfort' in this young adolescent population. This is in agreement with the observations by Broder et al. [49] among minority adolescents, and our findings on 'physical pain' and 'psychological discomfort' also agree with the observations by Ferreira et al. [47] in Brazilian schoolchildren, thus suggesting that some dimensions from these domains of OHRQoL frequently affect adolescents.

### Construct validity of the OHIP-Sp

The OHIP-Sp exhibited adequate convergent validity, in agreement with studies conducted using other versions of the Oral Health Impact Profile among adolescents [23,49].

A potential limitation of this study to assess discriminative validity is the lack of inclusion of a common pain-related dental health outcome such as caries, which could be a better oral health outcome to distinguish between groups of adolescents with known differences in dental health. The oral health outcomes used in this study are usually considered in studies among adults [53,54] but not in studies conducted among adolescents [23,49], in which the occurrence of tooth loss and periodontal disease is expected to be low. Nevertheless, the results of the assessment of discriminative validity using Mann Whitney statistics suggest that OHIP-Sp is suitable to distinguish between groups with and without oral conditions such as clinical attachment loss and tooth loss among adolescents. The area under the ROC curves for the four outcomes tested are not impressive and challenge the application of statistical testing for the assessment of discriminative validity.

The ROC curve areas for different severity levels of clinical attachment loss and increasing extent of tooth loss demonstrated that OHIP-Sp is suitable to discriminate subjects with increasing severity and/or extent of these dental outcomes.

### Internal consistency of the OHIP-Sp

The values for internal consistency estimated with Cronbach's alpha relate to OHIP scores obtained for an specific study group rather than to the instrument itself [55]. This means that the numerical size of Cronbach's alpha is significantly influenced by the degree of disease variation in the study group used to test the instrument. The Cronbach's alpha coefficients for internal consistency found in this study were slightly lower than those observed by Broder et al., [49] for disadvantaged adolescents, and similar to those obtained by Soe et al., [23] for Myanmar adolescents with low oral disease experience. The population in which the OHIP-Sp was tested represents one of the most demanding situations for the instrument. Our observation that OHIP-Sp did in fact capture oral health impacts when used in a young population with a low periodontal disease burden and very limited tooth loss testifies to the usefulness of the instrument. While it may be noted that the recommendation of Cronbach's alpha > 0.70 for sufficient internal consistency [42] was reached only for one of the domains and for the total summary score, it is also clear that most other domains were approaching this limit. Moreover, higher estimates for internal consistency are likely to be found if the instrument is applied to (older) study groups with more disease experience.

Clearly, further studies of the properties of OHIP-Sp should include testing of the questionnaire in older populations and in populations with a higher disease burden/disease variation; as well as the inclusion of caries as a dental outcome. Additional aspects of the instrument that should be assessed are the use of test-retest reliability exercises to evaluate the stability of the test; and the assessment of the responsiveness of OHIP-Sp to changes in oral health conditions.

## Conclusion

The OHIP-Sp revealed suitable convergent and discriminative validity and appropriate internal consistency.

## Abbreviations

OHIP Oral Health Impact Profile

OHIP-Sp Spanish version of the OHIP

OHRQoL Oral Health Related Quality of Life

NUG Necrotizing ulcerative gingival lesions

CAL Clinical attachment loss

## Competing interests

The author(s) declare that they have no competing interests.

## Authors' contributions

RL conceived and designed the study, collected the data, performed the statistical analysis, the interpretation of the data, and the manuscript drafting.

VB conceived and designed the study, and assisted in the collection, analysis, and interpretation of the data. Both authors reviewed, edited, and approved the manuscript.

## Pre-publication history

The pre-publication history for this paper can be accessed here:



## Supplementary Material

Additional file 1 Spanish version of the Oral Health Impact Profile (OHIP-Sp). 49 items OHIP questionnaire in Spanish. Click here for file

## References

[B1] Murray H, Locker D, Mock D, Tenenbaum HC (1996). Pain and quality of life in patients referred to a craniofacial pain unit. J Orofacial Pain.

[B2] Kressin NR (1996). Symposium on self-reported assessments of oral health outcomes. Introduction. J Dent Educ.

[B3] Slade GD (1996). Reactor paper. J Dent Educ.

[B4] Locker D (1996). Applications of self-reported assessment of oral health outcomes. J Dent Educ.

[B5] Slade GD (1998). Assessing change in quality of life using the Oral Health Impact Profile. Community Dent Oral Epidemiol.

[B6] Allen PF, McMillan AS (1999). The impact of tooth loss in a denture wearing population: an assessment using the Oral health impact profile. Community Dent Health.

[B7] Nuttall NM, Steele JG, Pine CM, White D, Pitts NB (2001). The impact of oral health on people in the UK in 1998. Br Dent J.

[B8] Locker D, Matear D, Stephens M, Jokovic A (2002). Oral health-related quality of life of a population of medically compromised elderly people. Community Dent Health.

[B9] McGrath C, Bedi R (2002). Measuring the impact of oral health on life quality in two national surveys-functionalist versus hermeneutic approaches. Community Dent Oral Epidemiol.

[B10] John MT, LeResche L, Koepsell TD, Hujoel P, Miglioretti DL, Micheelis W (2003). Oral health-related quality of life in Germany. Eur J Oral Sci.

[B11] Heydecke G, Locker D, Awad MA, Lund JP, Feine JS (2003). Oral and general health-related quality of life with conventional and implant dentures. Community Dent Oral Epidemiol.

[B12] Llewellyn CD, Warnakulasuriya S (2003). The impact of stomatological disease on oral health-related quality of life. Eur J Oral Sci.

[B13] McMillan AS, Pow EH, Leung WK, Wong MCM, Kwong DLW (2004). Oral health-related quality of life in southern Chinese following radiotherapy for nasopharyngeal carcinoma. J Oral Rehabil.

[B14] Gilbert GH, Meng X, Duncan RP, Shelton BJ (2004). Incidence of tooth loss and prosthodontic dental care: effects on chewing difficulty onset, a component of oral health-related quality of life. J Am Geriatr Soc.

[B15] Needleman I, McGrath C, Floyd P, Biddle A (2004). Impact of oral health on the life quality of periodontal patients. J Clin Periodontol.

[B16] Steele JG, Sanders AE, Slade GD, Allen PF, Lahti S, Nuttall N, Spencer AJ (2004). How do age and tooth loss affect oral health impacts and quality of life? a study comparing two national samples. Community Dent Oral Epidemiol.

[B17] John MT, Koepsell TD, Hujoel P, Miglioretti DL, LeResche L, Micheelis W (2004). Demographic factors, denture status and oral health-related quality of life. Community Dent Oral Epidemiol.

[B18] John MT, Hujoel P, Miglioretti DL, LeResche L, Koepsell TD, Micheelis W (2004). Dimensions of Oral-health-related quality of life. J Dent Res.

[B19] Okunseri C, Chattopadhyay A, Lugo RI, McGrath C (2005). Pilot survey of oral health-related quality of life: a cross-sectional study of adults in Benin City, Edo State Nigeria. BMC Oral Health.

[B20] Slade GD, Spencer AJ (1994). Development and evaluation of the oral health impact profile. Community Dent Health.

[B21] Locker D (1988). Measuring oral health: a conceptual framework. Community Dent Health.

[B22] Slade GD (1997). Derivation and validation of a short-form oral health impact profile. Community Dent Oral Epidemiol.

[B23] Soe KK, Gelbier S, Robinson PG (2004). Reliability and validity of two oral health related quality of life measures in Myanmar adolescents. Community Dent Health.

[B24] Locker D, Jokovic A, Clarke M (2004). Assessing the responsiveness of measures of oral health-related quality of life. Community Dent Oral Epidemiol.

[B25] Allen PF, McMillan AS, Locker D (2001). An assessment of sensitivity to change of the Oral Health Impact Profile in a clinical trial. Community Dent Oral Epidemiol.

[B26] Allison P, Locker D, Jokovic A, Slade G (1999). A cross-cultural study of oral health values. J Dent Res.

[B27] Lopez R, Fernández O, Jara G, Baelum V (2001). Epidemiology of clinical attachment loss in adolescents. J Periodontol.

[B28] Lopez R, Fernández O, Jara G, Baelum V (2002). Epidemiology of necrotizing ulcerative gingival lesions in adolescents. J Periodont Res.

[B29] Lopez R (2003). Periodontitis in adolescents. Studies among Chilean high school students.

[B30] Lopez R, Fernández O, Baelum V (2006). Social gradients in periodontal disease among adolescents.. Community Dent Oral Epidemiol.

[B31] Lopez R, Baelum V (2006). Gender differences in tooth loss among Chilean adolescents: Socioeconomic and behavioral correlates.. Acta Odontol Scand.

[B32] Hutchinson A, Bentzen N and König-Zahn C, European Research Group on Health Outcomes (1996). Cross cultural health outcome assessment; A user's guide.

[B33] Lopez R, Baelum V (2004). Necrotizing ulcerative gingival lesions and clinical attachment loss. Eur J Oral Sci.

[B34] Allen PF, McMillan AS, Walshaw D, Locker D (1999). A comparison of the validity of generic- and disease-specific measures in the assessment of oral health-related quality of life. Community Dent Oral Epidemiol.

[B35] Robinson PG, Gibson B, Khan FA, Birnbaum W (2003). Validity of two oral health-related quality of life measures. Community Dent Oral Epidemiol.

[B36] Larsson P, List T, Lundström I, Marcusson A, Ohrbach R (2004). Reliability and validity of a Swedish version of the Oral Health Impact Profile (OHIP-S). Acta Odontol Scand.

[B37] Saub R, Locker D, Allison P (2005). Derivation and validation of the short version of the Malaysian Oral Health Impact Profile. Community Dent Oral Epidemiol.

[B38] de Oliveira BH, Nadanovsky P (2005). Psychometric properties of the Brazilian version of the Oral Health Impact Profile- short form. Community Dent Oral Epidemiol.

[B39] StataCorp., stat (2005). StataCorp. Statistical Software: Release 9.0.

[B40] Hanley JA, McNeil BJ (1982). The meaning and use of the area under a Receiver Operating Characteristic (ROC) curve. Radiology.

[B41] Lee WC (1999). Probabilistic analysis of global performances of diagnostic tests: interpreting the Lorenz curve-based summary measures. Stat Med.

[B42] Bland JM, Altman DG (1997). Cronbach's alpha. Br Med J.

[B43] Cronbach LJ (1951). Coefficient alpha and the internal reliability of tests. Psychometrika.

[B44] Guillemin F, Bombardier C, Beaton D (1993). Cross-cultural adaptation of health-related quality of life measures: literature review and proposed guidelines. J Clin Epidemiol.

[B45] Beaton DE, Bombardier C, Guillemin F, Ferraz MB (2000). Guidelines for the process of cross-cultural adaptation of self-report measures. Spine.

[B46] Massof RW (2004). Likert and Guttman scaling of visual function rating scale questionnaires. Ophthalmic Epidemiol.

[B47] Ferreira CA, Loureiro CA, Araujo VE (2004). Psycometrics properties of subjective indicator in children. Rev Saude Publica.

[B48] de Oliveira CM, Sheiham A (2004). Orthodontic treatment and its impact on oral health-related quality of life in Brazilian adolescents. J Orthod.

[B49] Broder HL, Slade G, Caine R, Reisine S (2000). Perceived impact of oral health conditions among minority adolescents. J Public Health Dent.

[B50] John MT, Patrick DL, Slade GD (2002). The German version of the Oral Health Impact Profile - translation and psychometric properties. Eur J Oral Sci.

[B51] Wong MCM, Lo ECM, McMillan AS (2002). Validation of a Chinese version of the Oral Health Impact Profile (OHIP). Community Dent Oral Epidemiol.

[B52] McMillan AS, Wong MCM, Lo ECM, Allen PF (2003). The impact of oral disease among the institutionalized and non-institutionalized elderly in Hong Kong. J Oral Rehabil.

[B53] Ekanayake L, Perera I (2004). The association between clinical oral health status and oral impacts experienced by older individuals in Sri Lanka. J Oral Rehabil.

[B54] Ng SKS, Leung WK (2006). Oral health-related quality of life and periodontal status. Community Dent Oral Epidemiol.

[B55] Yu CH (2001). An introduction to computing and interpreting Cronbach Coefficient Alpha in SAS. Proceedings of the 26th SAS User Group International Conference.

