# Supplementary material for: Spanish version of the Oral Health Impact Profile (OHIP-Sp)
Source: BMC Oral Health. 2006 Jul 7;6:11. doi: 10.1186/1472-6831-6-11 (PMC1534011; doi:10.1186/1472-6831-6-11)
Supplement: Additional file 1 — Spanish version of the Oral Health Impact Profile (OHIP-Sp). 49 items OHIP questionnaire in Spanish. [file 1472-6831-6-11-S1.doc]

Spanish version of the Oral Health Impact Profile (OHIP-Sp)

**Limitación funcional**

1 ¿Has tenido dificultades mordiendo algún alimento por problemas con tus dientes, boca o prótesis?

2 ¿Has tenido problemas pronunciando alguna palabra por problemas con tus dientes, boca o prótesis?

3 ¿Has notado un diente que no se ve bien?

4 ¿Has sentido que tu apariencia ha sido afectada por problemas con tus dientes, boca o prótesis?

5 ¿Has sentido que tu aliento se ha deteriorado por problemas con tus dientes, boca o prótesis?

6 ¿Has sentido que tu sensación de sabor ha empeorado por problemas con tus dientes, boca o prótesis?

7 ¿Has retenido alimento en tus dientes o prótesis?

8 ¿Has sentido que tu digestión ha empeorado por problemas con tus dientes, boca o prótesis?

9 ¿Has sentido que tus prótesis no ajustan apropiadamente?

**Dolor físico**

10 ¿Has tenido molestias dolorosas en tu boca?

11 ¿Has tenido dolor en los maxilares?

12 ¿Has tenido dolor de cabeza por problemas con tus dientes, boca o prótesis?

13 ¿Has tenido dientes sensibles, por ejemplo debido a calor o alimentos o líquidos fríos?

14 ¿Has tenido dolor de dientes?

15 ¿Has tenido dolor de encías?

16 ¿Has encontrado inconfortable comer algún alimento por problemas con tus dientes, boca o prótesis?

17 ¿Has tenido áreas dolorosas en tu boca?

18 ¿Has tenido prótesis inconfortables?

**Disconfort psicológico**

19 ¿Has estado preocupado por problemas dentales?

20 ¿Has sido consciente de ti mismo por tus dientes, boca o prótesis?

21 ¿Los problemas dentales te han hecho miserable?

22 ¿Has sentido disconfort sobre la apariencia de tus dientes, boca o prótesis?

23 ¿Te has sentido tenso/a por problemas con tus dientes, boca o prótesis?

**Incapacidad física**

24 ¿Ha sido poco clara la forma en que tú hablas por problemas con tus dientes, boca o prótesis?

25 ¿La gente ha malentendido algunas de tus palabras por problemas con tus dientes, boca o prótesis?

26 ¿Has sentido que hay menos sabor en tus alimentos por problemas con tus dientes, boca o prótesis?

27 ¿Has sido incapaz para cepillar tus dientes apropiadamente por problemas con tus dientes, boca o prótesis?

28 ¿Has tenido que evitar comer algunos alimentos por problemas con tus dientes, boca o prótesis?

29 ¿Tu dieta ha sido insatisfactoria por problemas con tus dientes, boca o prótesis?

30 ¿Has sido incapaz de comer con tus prótesis por problemas con ellas?

31 ¿Has evitado sonreír por problemas con tus dientes, boca o prótesis?

32 ¿Has tenido que interrumpir comidas por problemas con tus dientes, boca o prótesis?

**Incapacidad psicológica**

33 ¿Tu sueño ha sido interrumpido por problemas con tus dientes, boca o prótesis?

34 ¿Has estado molesto por problemas con tus dientes, boca o prótesis?

35 ¿Has encontrado difícil relajarte por problemas con tus dientes, boca o prótesis?

36 ¿Te has sentido deprimido por problemas con tus dientes, boca o prótesis?

37 ¿Se ha afectado tu concentración por problemas con tus dientes, boca o prótesis?

38 ¿Has estado un poco avergonzado por problemas con tus dientes, boca o prótesis?

**Incapacidad social**

39 ¿Has evitado salir por problemas con tus dientes, boca o prótesis?

40 ¿Has sido menos tolerante con tu pareja o familia por problemas con tus dientes, boca o prótesis?

41 ¿Has tenido problemas relacionándote con otra gente por problemas con tus dientes, boca o prótesis?

42 ¿Has estado un poco irritable con otra gente por problemas con tus dientes, boca o prótesis?

43 ¿Has tenido dificultades haciendo tu trabajo habitual por problemas con tus dientes, boca o prótesis?

**En desventaja**

44 ¿Has sentido que tu salud general ha empeorado por problemas con tus dientes, boca o prótesis?

45 ¿Has sufrido cualquier pérdida financiera por problemas con tus dientes, boca o prótesis?

46 ¿Has sido incapaz de disfrutar mucho la compañía de otra gente por problemas con tus dientes, boca o prótesis?

47 ¿Has sentido que la vida en general fue menos satisfactoria por problemas con tus dientes, boca o prótesis?

48 ¿Has sido totalmente incapaz de funcionar por problemas con tus dientes, boca o prótesis?

49 ¿Has sido incapaz de trabajar a tu capacidad total por problemas con tus dientes, boca o prótesis?
